# Classifying Dementia Using Local Binary Patterns from Different Regions in Magnetic Resonance Images

**DOI:** 10.1155/2015/572567

**Published:** 2015-03-22

**Authors:** Ketil Oppedal, Trygve Eftestøl, Kjersti Engan, Mona K. Beyer, Dag Aarsland

**Affiliations:** ^1^Department of Electrical Engineering and Computer Science, University of Stavanger, 4036 Stavanger, Norway; ^2^Centre for Age-Related Medicine, Stavanger University Hospital, Stavanger, Norway; ^3^Department of Radiology and Nuclear Medicine, Oslo University Hospital, Oslo, Norway; ^4^Department of Life Sciences and Health, Faculty of Health Sciences, Oslo and Akershus University College of Applied Sciences, Oslo, Norway; ^5^Alzheimer's Disease Research Centre, Karolinska Institutet (KI), Stockholm, Sweden

## Abstract

Dementia is an evolving challenge in society, and no disease-modifying treatment exists. Diagnosis can be demanding and MR imaging may aid as a noninvasive method to increase prediction accuracy. We explored the use of 2D local binary pattern (LBP) extracted from FLAIR and T1 MR images of the brain combined with a Random Forest classifier in an attempt to discern patients with Alzheimer's disease (AD), Lewy body dementia (LBD), and normal controls (NC). Analysis was conducted in areas with white matter lesions (WML) and all of white matter (WM). Results from 10-fold nested cross validation are reported as mean accuracy, precision, and recall with standard deviation in brackets. The best result we achieved was in the two-class problem NC versus AD + LBD with total accuracy of 0.98 (0.04). In the three-class problem AD versus LBD versus NC and the two-class problem AD versus LBD, we achieved 0.87 (0.08) and 0.74 (0.16), respectively. The performance using 3DT1 images was notably better than when using FLAIR images. The results from the WM region gave similar results as in the WML region. Our study demonstrates that LBP texture analysis in brain MR images can be successfully used for computer based dementia diagnosis.

## 1. Introduction

*Dementia Is an Evolving Challenge*. As a result of increasing age, dementia is an evolving challenge in society. The annual health care costs related to dementia were estimated to $604 billion worldwide in 2010 [1]. Alzheimer's disease (AD) is the most common neurodegenerative dementia and accounts for 50–60% of people with dementia [2]. The classical neuropathological signs of AD are amyloid plaques and neurofibrillary tangles [3]. No efficient disease-modifying treatment for AD exists today. Dementia with Lewy-bodies (DLB) together with dementia associated with Parkinson's disease (PDD) account for 15–20% of people with dementia [2]. The defining pathological feature for these patients is Lewy-body degeneration in brain stem, forebrain, and limbic and cortical structures, and the DLB and PDD are therefore often combined into a Lewy-body dementia group (LBD) [4, 5]. However, the relationship between localization and density of Lewy-bodies with clinical dementia symptoms is not strong [6], suggesting that other pathologies contribute as well, such as AD pathology and vascular brain changes seen as white matter hyperintensities (WML) or lacunar infarcts, which may contribute to the clinical presentation of LBD. For example, vascular changes in the basal ganglia are common in the elderly and may cause parkinsonism and cognitive impairment [7].

*Early Diagnosis Is Important*. AD and LBD are very complex diseases making them difficult to be prevented, delayed, or cured. Current therapy focuses on many approaches, for example, helping patients maintain an acceptable mental functioning, managing typical behavioural changes, and slowing symptom progression. Early intervention is important, and the ability to identify these types of dementia and healthy controls early in the disease course may be essential for successful patient care. Differentiating between AD and LBD is also important since they differ in prognosis and response to drug treatment. Currently, the only available method to differentiate between AD and LBD is the dopamine transporter scan, which is expensive and not readily available at all centres. 

*Neuroimaging in Dementia.* Neuroimaging is an important tool for studying dementia and cognitive deterioration. Several excellent reviews are available [8–10]. In [11], Malloy et al. review available methods for quantitative imaging of white matter anatomy and pathology as well as recent findings in ageing and dementia. They state that computer aided quantification offers better statistical power compared to visual rating scales and that diffusion imaging is able to detect abnormalities not recognised in conventional acquisition sequences. Early detection of disease and relevant functional connections between brain areas are important benefits. 

*Computer Aided Diagnosis in Dementia*. Computer aided diagnosis (CAD) can be a helpful tool to pinpoint diagnosis early in the disease course in a cost-effective manner and unbiased to human inconsistencies [12]. Recent advances in the field have focused especially on AD and patients with mild cognitive impairment (MCI), which are considered a precursor to AD [13–16]. Less attention has been put into developing CAD systems for LBD. As mentioned above, LBD have high prevalence, and accurate clinical diagnosis depends on little available and expensive dopamine transporter scan and postmortem histology. Few papers report high accuracy discerning patients with AD and LBD or other dementias [17, 18]. A promising approach is reported in [19] where Lebedev et al. use sparse partial least squares (SPLS) classification of cortical thickness measurements reporting a sensitivity of 94.4 and a specificity of 88.89 discerning AD from LBD. 

*White Matter and White Matter Lesions in Dementia*. White matter (WM) comprises approximately half the brain volume and provides connectivity between the two brain hemispheres as well as ensuring efficient transfer of neural activity complementing information processing in the gray matter (GM). WM neuropathology is often diffuse and affects many neuronal networks which can be disturbed simultaneously resulting in a multidomain syndrome. In [20], Filley emphasizes the contribution of white matter disease (WMD) in mild cognitive dysfunction, cognitive ageing, and dementia. Bartzokis [21] proposes a hypothesis for AD called the* “myelin model”* where axonal transport disruption, formation of axonal swellings, neuritic plaques, and proteinaceous deposits such as A*β* and tau are by-products of homeostatic myelin repair processes. Gunning-Dixon et al. [22] review results of MRI studies of white matter changes that occur with normal ageing and the relationship of age-associated changes in white matter to age-related declines in cognitive abilities.

White matter lesions or white matter hyperintensities (WML) are among the neuroimaging expressions of cerebral small-vessel disease and are associated with various disturbances with poor prognosis [23]. WML are localized areas of increased signal intensities in the white matter of the brain visible on T2-weighted MR images. The underlying pathology of WML is heterogeneous, ranging from mild demyelination to incomplete subcortical infarctions. They are typically seen around the ventricles (periventricular WML), but also as focal lesions in the deep white matter. In the elderly, WML usually represent small-vessel cerebrovascular disease (CVD) [24]. WML becomes more abundant with increasing age in healthy subjects [25], but they are also found to be associated with AD [26] and other dementias [27, 28]. Clinical symptoms associated with WML include gait disturbances [29], depression [27, 30], and cognitive impairment [31], although the exact mechanisms are not fully understood. In [32], Tuladhar et al. concludes that cortical changes mediated by WML and vascular risk factors might lead to cognitive decline and dementia. Muñoz Maniega et al. [33] write that age-related deterioration of normal appearing white matter (NAWM) is strongly associated with the severity of WML, indicating that WML is important in dementia research. Fujishima et al. conclude in [34] that mild cognitive impairment, poor episodic memory, and late-life depression are associated with cerebral cortical thinning and WML. 

*Texture Analysis in Neuroimaging*. Harrison [35] extensively reviews the use of texture analysis in a clinical context, analysing MR images in non-Hodgkin lymphoma, mild traumatic brain injury, and multiple sclerosis. She concludes that “non visible lesions and physiological changes as well as visible focal lesions of different aetiologies could be detected and characterized by texture analysis of routine clinical 1.5 Tesla scans.” The application of texture analysis in a machine learning (ML) environment has shown success in discerning different dementias from each other and from healthy controls. Freeborough and Fox reported a classification rate of 91% discerning AD from healthy controls using measures from a spatial gray-level dependent method applied in a stepwise discriminant analysis approach in [36]. de Olivieira et al. [37] found statistical significant differences in gray level cooccurrence matrix measurements in subjects with mild AD, amnestic mild cognitive impairment (aMCI), and healthy controls. Zhang et al. [38] performed 3D texture analysis in MR images of the hippocampus and entorhinal cortex in AD patients and achieved a classification accuracy between 64.3% and 96.4%. Sivapriya et al. showed in [39] that texture analysis in brain MRI gave high classification accuracy in AD. As of the authors knowledge, the only paper considering texture analysis as an approach to distinguish AD, LBD, and NC is [40], where Kodama and Kawase performed discriminant analysis on features extracted from a cooccurrence matrix and a run-length matrix with an accuracy of 91.7%, 70.0%, and 88.0%, respectively.

Local binary pattern (LBP) was introduced by Ojala et al. [41, 42] as a texture descriptor. It is a simple yet very efficient texture operator which labels the pixels of an image by thresholding the neighbourhood of each pixel and considers the result as a binary number. Unay et al. [43] showed that the rotation invariant LBP is invariant to some common MRI artefacts which makes it a robust texture feature when used in brain MR image analysis. 

*Aims*. We have earlier shown that there were no differences in WML volume between patients with AD and LBD or between a combined dementia group (AD + LBD) and healthy controls in the DemVest study [44]. Now we want to test if the WML regions inherit textural information in an extent that can be used to classify dementia patients from normal controls and AD from LBD. As the detection of textural information in WML might not be dependent on an exact delineation of WML, we also want to test if a comparable classification accuracy can be achieved using all of WM as ROI, since WM segmentation is more available and only a 3DT1 MR image is needed which is commonly acquired in a clinical setting.

Earlier we have shown that using LBP texture analysis in WML regions in FLAIR MR images in a machine learning (ML) context can discern patients with dementia from healthy controls with high accuracy [45]. We want to test different types of LBP calculations together with a contrast measure (C) calculated from FLAIR and 3DT1 MR images from a cohort study (the DemWest and ParkWest study) and on a subset containing data from one scanner only.

Because of the challenging situation with imbalanced data having different numbers of subjects in the represented groups in the abovementioned cohorts, we want to test how the use of resampling of instances affects classification results. 

*Organisation of Paper*. The paper is organised as follows: Section 2 describes the data material, Section 3 describes the image preprocessing procedures followed by Section 4 which describes the image processing methods and Section 5 describing the experimental setup.  Section 6 reveals the results.  Section 7 discusses the results and ends the paper with a conclusion.

## 2. Material

### 2.1. Subjects

MR images of dementia subjects included in this study were drawn from the DemWest cohort, Stavanger, Norway, and MR images of the healthy controls from the ParkWest cohort, Stavanger, Norway. Inclusion and exclusion criteria can be found in [2] and [46], respectively. The dementia and healthy control subjects were matched for sex, age, and years of education.

The Regional Committee for Medical Research Ethics, Western Norway, approved the study. All participants signed informed consent to participate in the study after the study procedures had been explained in detail to the patient and a caregiver, usually the spouse or offspring.

### 2.2. MRI

The dementia patients were scanned at three different sites: Stavanger University Hospital, Stavanger, Norway, Haugesund Hospital, Haugesund, Norway, and Haraldsplass Deaconess Hospital, Bergen, Norway. A 1.5 T scanner was used in all three centres (Philips Intera in Stavanger and Haugesund and GE Signa Excite in Bergen), using the same scanner in each centre during the entire study period and a common study imaging protocol.

The NCs were scanned at four different sites. They were scanned on the same scanners as the patients in Stavanger and Haugesund. Additionally MR images of NC subjects were acquired from Sørlandet Hospital Arendal, Arendal, Norway (1.0T Philips Intera), and Unilabs, Bergen, Norway (1.5T Siemens Symphony).

After visual inspection, some patient scans were excluded due to either insufficient image quality, not having both FLAIR and T1 images for the patient, or movement and other artefacts.

A total of 73 mild dementia subjects, 57 with AD and 16 with LBD, had MRI scans of sufficient quality and were included in this study as well as 36 healthy controls. In [44], further clinical details as well as MR imaging parameters can be found.

To ensure high reliability between scans acquired at different centres and at different time points, three volunteers were scanned at all centres using the same scanners and protocols. Details of the procedure can be found in [44]. Cronbachs alpha between MR scanners at different centres was calculated based on total brain volume and was reported to be 0.958. Cronbachs alpha between two time points varied between 0.982 and 0.995, indicating excellent reliabilities both between centres and between different time points. Similar results were reported for the MR images of the NCs from the ParkWest study.

## 3. Image Preprocessing

### 3.1. Region of Interest Extraction

Two common approaches for MR image segmentation of the brain are tissue classification and template registration. In the tissue classification approach, voxels are assigned to a class based on the class voxel intensity distribution. In the template registration approach, a template image with predefined classes is warped to the actual MR image. In our study, WM partitions were segmented using the common functions in SPM8 on the T1 images. The procedure unifies a tissue segmentation approach with a template registration method; see [47] for further details.

WML segmentation was performed according to a method developed and previously published by Firbank et al. in Newcastle, England [48]. The method is based on determining a threshold value from the image gray scale intensity values and then classifying the hyperintense voxels as WML. Briefly, the nonbrain regions were removed from the T1 image, using the segmentation routines in the software package SPM5 [49]. After transforming to the image space of the FLAIR image, the segmented T1 image was used as a mask for scull stripping of the FLAIR image. Then the WML were segmented automatically on a slice-by-slice basis from the FLAIR images with the images in native space. A scale factor determined experimentally was multiplied by the mode of the histogram of pixel intensities for each image slice and used as a threshold value for WML segmentation. To explore the regional distribution of WML throughout the brain, a region of interest (ROI) template in standard MNI space [50] was used. This ROI template was transformed from MNI space to the image space (FLAIR) of each subject by use of the normalization routines in SPM5, and the volumes of WML in each ROI were calculated. The ROI map was based on the Brodmann template. Further details can be found in [51].

Because of the variability in MR image quality acquired from the different centres participating in this study, a scale factor that gave an overestimation of the lesion load in every subject was selected, and manual editing was then done to correct this by removing excess pixels using FSLView [52], a medical image-editing program being a part of the FSL software bundle. A medical doctor did the manual editing after training by a consultant radiologist who is experienced at evaluation of WML. We performed inter- and intrarater reliability testing between the two raters to ensure good quality. They both edited the same 10 data sets twice: once in the beginning to ensure good interrater reliability and a second time at the end to ensure that similar reliability still persisted and to evaluate intrarater reliability. Intraclass correlation coefficient (ICC) was 0.998 for interrater reliability and 0.964 for intrarater reliability.

## 4. Image Processing Methods

### 4.1. LBP

Ojala et al. [41, 42] introduced LBP as a texture operator. Since its discriminative power is high and at the same time computationally simple, LBP is a popular texture descriptor used in various applications and unifies traditionally divergent statistical and structural models of texture analysis. Adding an image contrast measure (*C*) calculating the local variance in the pixel neighbourhood, as well as varying the texture neighbourhood, enhances the discriminative power of the LBP feature even further. In [43], Unay et al. demonstrated that the rotation invariant LBP is invariant to some common MRI artefacts, that is, the bias field.

The derivation of the gray scale and rotation invariant texture operator LBP starts by defining texture *T* in a local neighbourhood of a monochrome texture image as the joint distribution of the gray levels of *P* (*P* > 1) image pixels:

(1) $$\begin{array}{c} T=t\left(g_{c},g_{0},\ldots ,g_{P-1}\right), \end{array}$$

where gray value *g*_*c*_ corresponds to the gray value of the center pixel of the local neighbourhood and *g*_*p*_ (*p* = 0,…, *P* − 1) corresponds to the gray value of *P* equally spaced pixels on a circle of radius *R* (*R* > 0) that form a circularly symmetric neighbour set. When the coordinates of *g*_*c*_ are (0,0), the coordinates of *g*_*p*_ are given by (−*R*sin(2*πp*/*P*), *R*cos⁡(2*πp*/*P*)) and the gray values of neighbours which do not fall exactly in the center of pixels are estimated by interpolation.

To achieve gray-scale invariance, the gray value of the center pixel (*g*_*c*_) is subtracted from the gray values of the circular symmetric neighbourhood *g*_*p*_ (*p* = 0,…, *P* − 1), giving

(2) $$\begin{array}{c} T=t\left(g_{c},g_{0}-g_{c},g_{1}-g_{c},\ldots ,g_{P-1}-g_{c}\right). \end{array}$$

By assuming that differences *g*_*p*_ − *g*_*c*_ are independent of *g*_*c*_ and thereby factorizing, we get

(3) $$\begin{array}{c} T\approx t\left(g_{c}\right)t\left(g_{0}-g_{c},g_{1}-g_{c},\ldots ,g_{P-1}-g_{c}\right). \end{array}$$

The distribution *t*(*g*_*c*_) describes the overall luminance of the image and is unrelated to local image texture and is removed. The approximated distribution

(4) $$\begin{array}{c} T\approx t\left(g_{0}-g_{c},g_{1}-g_{c},\ldots ,g_{P-1}-g_{c}\right) \end{array}$$

conveys many of the textural characteristics from the original.

By considering just the signs of the differences instead of their exact values, invariance with respect to gray-scale shifts is achieved:

(5) $$\begin{array}{c} T\approx t\left(s\left(g_{0}-g_{c}\right),s\left(g_{1}-g_{c}\right),\ldots ,s\left(g_{P-1}-g_{c}\right)\right), \end{array}$$

where

(6) $$\begin{array}{c} s\left(x\right)=\left\{\begin{array}{cc} 1, & x\geq 0\\ 0, & x<0. \end{array}\right. \end{array}$$

Each sign *s*(*g*_*p*_ − *g*_*c*_) is assigned a binomial factor 2^*p*^, such that *T* is transformed into a unique LBP_*P*,*R*_ number that characterizes the spatial structure of the local image texture:

(7) $$\begin{array}{c} {\text{LBP}}_{P,R}=\overset{P-1}{\underset{p=0}{\sum }}s\left(g_{p}-g_{c}\right)2^{P}. \end{array}$$

See also Figure 1.

To assign a unique identifier to each rotation invariant local binary pattern, LBP_*P*,*R*_^*ri*^ is defined as

(8) $$\begin{array}{c} {\text{LBP}}_{P,R}^{ri}=\min\left\{\text{ROR}\left({\text{LBP}}_{P,R},i\right)\;|\;i=0,1,\ldots ,P-1\right\}, \end{array}$$

where ROR(*x*, *i*) performs a circular bitwise right shift on the *P*-bit number *x* *i* times.

Certain local binary patterns are fundamental properties of texture. “Uniform” patterns are circular structures that contain very few spatial transitions. They function as templates for microstructures such as bright spot, flat area, dark spot, and edges of varying positive and negative curvature. The uniformity relates to the number of spatial transitions (i.e., bitwise 0/1 changes) in the LBP pattern; for example, 00000000_2_ and 11111111_2_ have a uniformity value *U*(“*pattern*”) of 0 and 00000011_2_ and 10000111_2_ of 1 and 2, respectively. Patterns that have a *U* value of at most 2 are designated as “uniform.” A gray-scale, rotation invariant, and uniform LBP texture operator are defined as follows:

(9) $$\begin{array}{c} {\text{LBP}}_{P,R}^{riu2}=\left\{\begin{array}{cc} \overset{P-1}{\underset{p=0}{\sum }}s\left(g_{p}-g_{c}\right), & \text{if}\;\;U\left({\text{LBP}}_{P,R}\right)\leq 2,\\ P+1, & \;\text{otherwise}, \end{array}\right. \end{array}$$

where

(10) ULBPP,R=sgP−1−gc−sg0−gc+∑p=1P−1sgp−gc−sgp−1−gc.

Superscript *riu*2 reflects the use of rotation invariant “uniform” patterns that have a *U* value of at most 2. By definition, exactly *P* + 1 “uniform” binary patterns can occur in a circularly symmetric neighbour set of *P* pixels whereas the “nonuniform” patterns are grouped under a miscellaneous label (*P* + 1).

### 4.2. Contrast

The LBP_*P*,*R*_^*ri*^ and LBP_*P*,*R*_^*riu*2^ operators are excellent measures of spatial patterns but discard contrast. If gray-scale invariance is not required, the contrast (*C*) of local image texture can be measured with a rotation invariant measure of local variance defined as

(11) $$\begin{array}{c} {\text{VAR}}_{P,R}=\frac{1}{P}\overset{P-1}{\underset{p=0}{\sum }}{\left(g_{p}-\mu \right)}^{2},\;\text{where}\;\;\mu =\frac{1}{P}\overset{P-1}{\underset{p=0}{\sum }}g_{p}, \end{array}$$

which is invariant against shifts in gray-scale.

The LBP and *C* values are calculated for every voxel in the specified region of interest creating an LBP- and a *C*-valued image. Typically, the LBP and *C* values are collected and represented as a histogram for each instance in the data set. The histogram can be used as a vector of features. Other approaches include calculating new features from the histogram.

## 5. Proposed Method and Experimental Setup

### 5.1. Overview of Proposed Method

**Figure 2:**
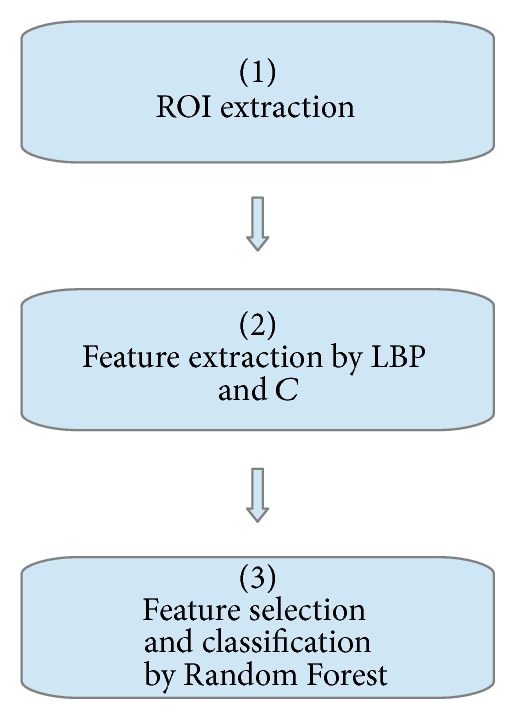
Overview of proposed method. See Section 5.1 for details.

A computer based system for classification of AD, LBD, and healthy controls based on texture analysis was applied. Firstly, the two regions of interest, WML and WM, were extracted from the MR images. The WM regions were segmented using common functions in SPM8 and the WML were segmented from the FLAIR images using the thresholding technique proposed by Firbank et al. [48], as briefly described in Section 3.1. See also Block 1 in Figure 2.

Secondly, rotation invariant 2D LBP and contrast were extracted voxel-wise for the two different ROIs using different combinations of neighbourhood radii and number of samples. The 2D LBP and contrast texture analysis were done both on the FLAIR and the T1 MR images (see Section 4.1 for information concerning the calculation of the LBP texture feature and Section 4.2 for the contrast measure and Block 2 of Figure 2). Statistical features were calculated from all the LBP and *C* values in each ROI were then calculated.

Eventually, a combined feature selection and classification procedure were applied using a Random Forest [53] classifier together with a nested cross validation procedure [54]. See Block 3 in Figure 2.

### 5.2. Texture Feature Extraction

**Figure 3:**
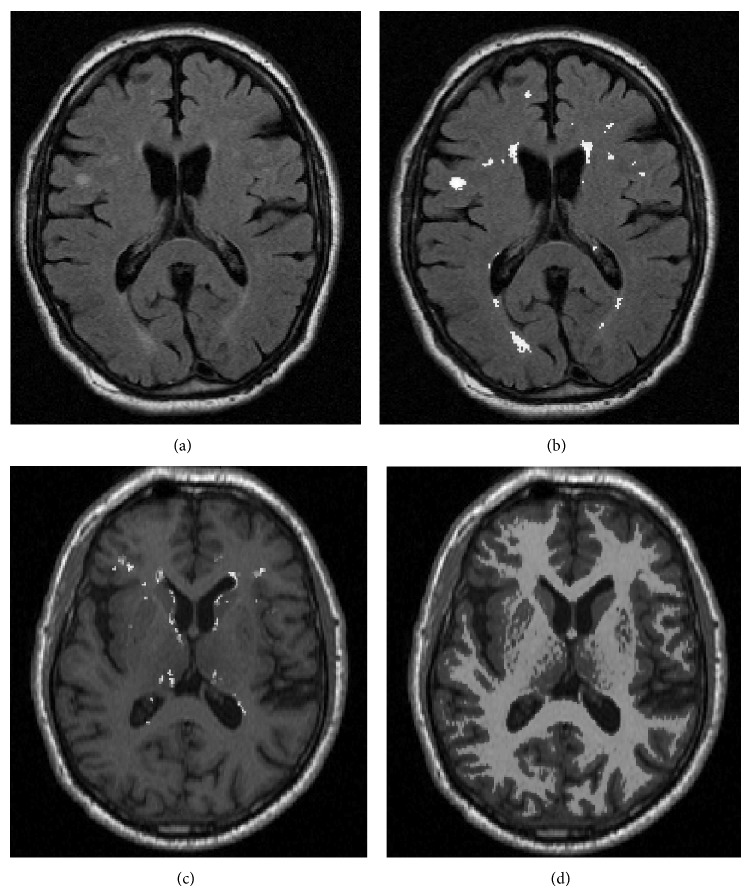
Overview of MR images and the ROIs used for feature extraction. (a) in the top left corner shows an example of an axial FLAIR MR image. The white matter lesions are possible to see as hyperintense areas. (b) in the top right shows the segmented voxels labelled as WML overlayed on the FLAIR MR image seen in (a). (c) in the bottom left corner shows the segmented WML voxels, found from the corresponding FLAIR, overlayed on the T1 MR image. (d) in the bottom right corner shows the segmented WM voxels overlayed on the T1 MR image.

**Figure 4:**
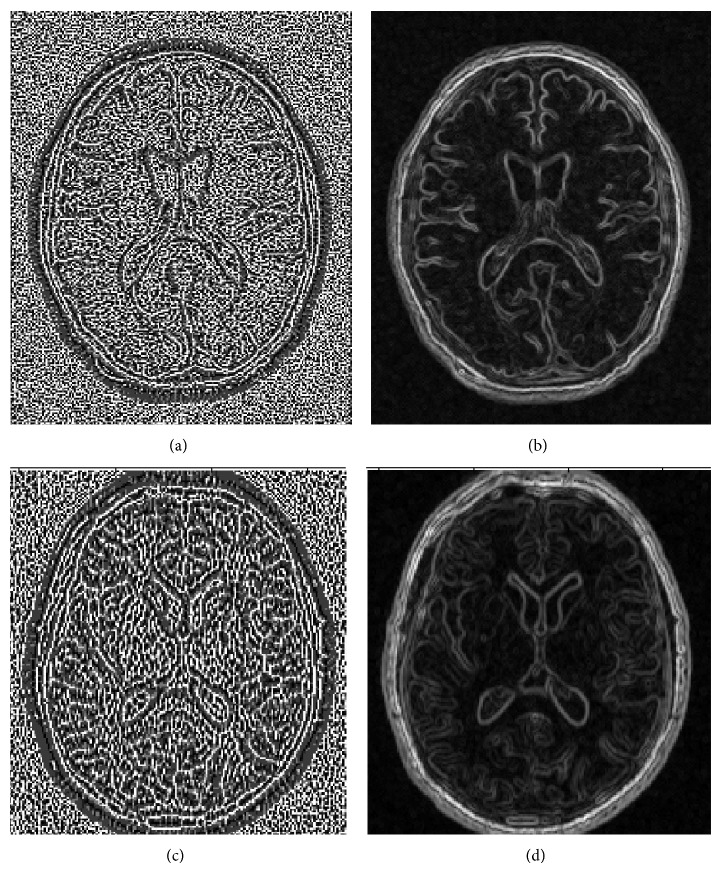
Overview of LBP texture- and contrast-valued images based on the FLAIR and T1 images. (a) in the top left corner shows an example of an LBP-valued image calculated from a FLAIR MR image. (b) in the top right corner shows a contrast-valued image calculated from FLAIR MRI. (c) in the bottom left corner shows an LBP-valued image calculated from a T1 image. (d) in the bottom right corner shows a contrast-valued image calculated from a T1 MR image.

For the 2D texture analysis approach, the LBP values as well as the *C* measure were calculated from every voxel in the selected ROI and MR image type for all subjects in the data set using Matlab [55]. Three different combinations of neighbourhood radius (*R*) and number of samples (*P*), namely, *R* = 1 and *P* = 8, *R* = 2 and *P* = 12, and *R* = 4 and *P* = 16, were used. Mean, standard deviation, variation, median, interquartile range, entropy, skewness, and kurtosis of the ROI-wise collected LBP and *C* values were calculated to be used as a descriptor of the distributions of the LBP and *C* values. These features were subjected to further selection and classification resulting in 8 features for each of the three combinations of *R* and *P* for both LBP and *C* resulting in a total of 48 features for each subject. See Figure 3 for an example of the FLAIR and T1 MR images and the WML segmentation results. See also Figure 4 for an example of LBP- and *C*-valued images based on the FLAIR and T1 MR images.

### 5.3. Feature Selection and Classification

**Figure 5:**
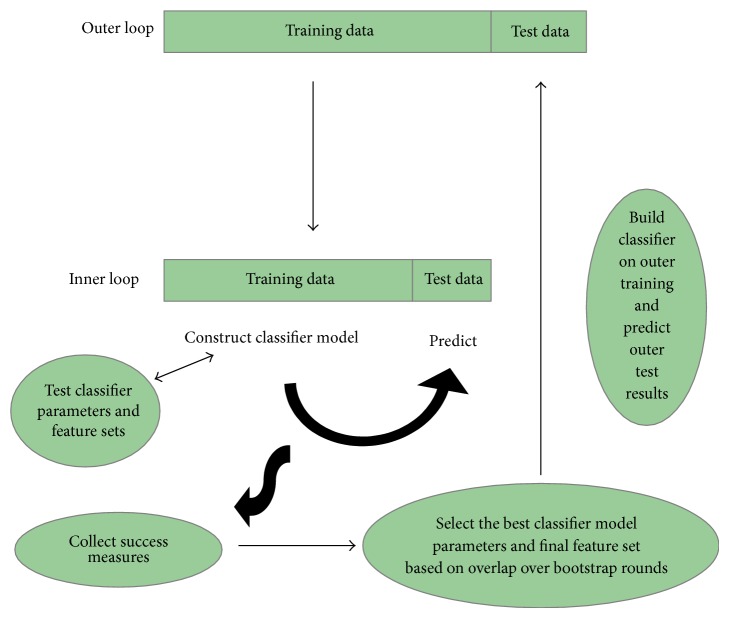
Nested cross validation: in the inner loop, the performance of different sets of classifier parameters and features is estimated based on a bootstrap cross validation. The optimal classifier parameters and features are selected based on the performance evaluation over several bootstrap rounds. In the outer loop, model performance of the optimized classifier parameters and features is evaluated on the hold-out test set in the outer loop. The outer loop is repeated several times, every time with potentially different classifier parameters and features.

A challenge in the developed machine learning task was the high number of features calculated compared to the size of the data set. Since the data were collected in a cohort study and thereby inexpedient to expand, a method for feature subset selection was needed. A method combining feature selection and classification using two nested cross validation loops together with a Random Forest classifier was chosen: an inner CV scheme for classification parameter and feature selection and an outer CV scheme for final model testing; see Figure 5 for details. Such an approach prevents the improper procedure of using the complete data set for supervised feature selection ahead of using cross validation for performance evaluation. The latter approach would give an overly optimistic result.

Image data were selected with stratification during bootstrap rounds in the cross validation procedure, meaning that the relative representation of instances in each class was kept intact.

Feature selection and classification were done using a 10-tree Random Forest classifier and 10-fold nested cross validation for performance evaluation. Search method was* best first*, start set with* no attributes*, search direction* forward*, stale search* after five node expansions*, subset evaluation* f-measure*, and number of folds for accuracy estimation was* 10*.

Pretesting was done using different classifiers, including support vector machines, Random Forests, and a Bayesian network classifier. The Random Forest classifier outperformed the other classifiers, and thus all experiments presented in this work are conducted using Random Forest classifiers.

**Figure 6:**
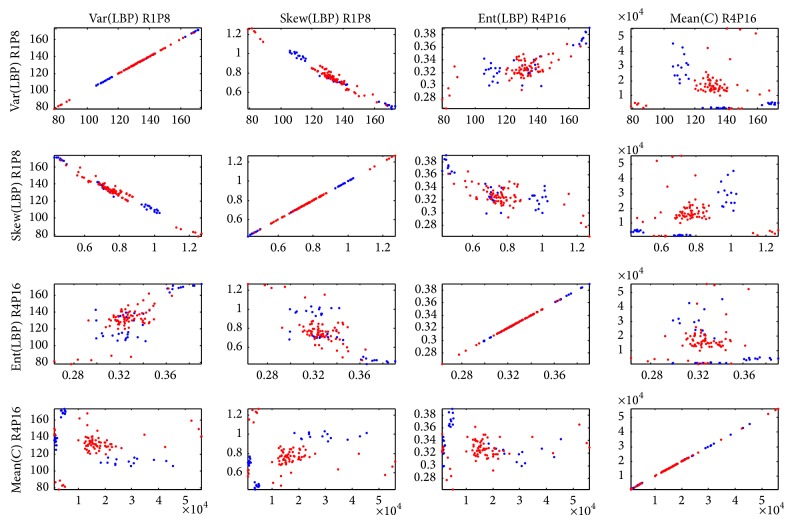
Matrix of scatter plots displaying the five selected features pairwise against each other. Blue depicts normal controls and red depicts dementia.

To give a fairly acceptable graphic display of the selected features, the feature and model parameter selection were eventually performed on the complete data set and a matrix of scatter plots displaying the five selected features pairwise against each other was made; see Figure 6. Note that this was only done for the sake of practical graphical display and as an example of which features that typically would be selected.

Random Forest is a classifier based on ensembles of decision trees developed by Breiman [53]. Many decision trees are built using bootstrap aggregation (bagging) and randomized feature subset sampling where the mode of the classes output by individual trees is voted for.

Three separate tests were explored: a three-class approach classifying NC versus AD versus LBD, a two-class approach classifying a NC versus a combined dementia group (AD + LBD), and another two-class approach classifying AD versus LBD.

### 5.4. Classification Accuracy

Precision for a class is the fraction of instances that are correctly classified to all instances that are classified as this class and is also known as positive predictive value. Recall for a class is the fraction of instances that are correctly classified to all the instances that really belong to this class and is also known as true positive rate or sensitivity.

In the context of a two-class problem where one class is the positive class and the other is the negative class, the true positives (TP) are the instances that are correctly classified as belonging to the positive class and the false positives (FP) are the instances that are classified as the positive class but really belong to the negative class. The true negatives (TN) and false negatives (FN) can be explained similarly. An overview of results can be presented as a confusion matrix, see Table 1.

Precision is then defined as Precision = TP/(TP + FP) and recall is defined as Recall = TP/(TP + FN). Total accuracy (*T*), precision (*P*), and recall (*R*) were calculated for each of the ten folds in the cross validation procedure resulting in ten values for each (*T*_1_, *T*_2_,…, *T*_10_), (*P*_1_, *P*_2_,…, *P*_10_), and (*R*_1_, *R*_2_,…, *R*_10_). Empirical mean over the ten values was calculated using the equation below:

(12) $$\begin{array}{c} m_{x}=\frac{1}{n}\overset{n}{\underset{k=1}{\sum }}x_{k},\;\text{where}\;\;0\leq x_{k}\leq 1,\;\;0\leq m_{x}\leq 1, \end{array}$$

where *x* is either *T*, *P*, or *R* and *n* = 10. The empirical standard deviation was calculated as below:

(13) sx=1n−1∑k=1nxk−mx,k21/2where  0≤xk≤1, 0≤sx≤1,

where *x* is either *T*, *P*, or *R*, *n* = 10, and *m*_*x*_ is defined as in (12). *T*, *P*, and *R* are reported as *m*(*s*) over 10-fold CV.

### 5.5. Imbalanced Data Set

The data set used in this study was drawn from a cohort. A common drawback is the problem of imbalanced data, meaning that the data set contains groups of different sizes. Typically, machine learning algorithms will perform poorly under such circumstances. As a measure to prevent such a problem, a resampling technique was used to even out the sizes of the groups.

All tests were done using the Synthetic Minority Oversampling Technique (SMOTE) [56, 57] to resample data, such that all classes had the same number of instances and are similar to the largest class in the original data. Similar tests were done without resampling as well, and, in all of the cases, the classification accuracy for the LBD class improved using SMOTE at the expense of classification accuracy for the other classes. Total accuracy was either improved or at least preserved. In conclusion, balancing out the number of instances in each class in the data set balanced out the classification performance for each class as well.

## 6. Results

### 6.1. Three-Class Problem: NC versus AD versus LBD

Results for the three-class problem with class 0 being NC, class 1 being AD, and class 2 being LBD are shown in detail in Table 2.  *T* is the total accuracy for all three classes. *P*0 is the precision for the NC group, *P*1 is the precision for the AD group, *P*2 is the precision for the LBD group, *R*0 is the recall for the NC group, *R*1 is the recall for the AD group, and *R*2 is the recall for the LBD group.

The first test named FLAIR-WML_*ri*_ indicates that the FLAIR MR image was used for calculation of LBP and *C*, that WML was the ROI, and that the rotational invariant variant of the LBP feature was used. The second test named T1WML_*ri*_ indicates that the T1 MR images were used for calculation of LBP and *C*, that WML was the ROI, and that the rotational invariant variant of the LBP feature was used. The third test named T1WM_*ri*_ indicates that the T1 MR images were used for calculation of the LBP and *C*, that the WM was the ROI, and that the rotational invariant variant of the LBP feature was used.

The total accuracy showed great variation throughout the different tests ranging from 0.6 (0.13) to 0.87 (0.08). The performance increased considerably when calculating the LBP and *C* features from the T1 MR image as compared to the FLAIR MR image. The classification performance proved best in the T1 case and when WML was used as ROI.

For comments on the T1WML_svg,*ri*_ test, see Section 6.4.

### 6.2. Two-Class Problem: NC versus AD + LBD

Results for the two-class problem with class 0 being NC and class 1 being AD and LBD together are shown in detail in Table 3.  *T* is the total accuracy for the two classes. *P*0 is the precision for the NC group and *P*1 is the precision for the combined AD and LBD group; *R*0 is the recall for the NC group and *R*1 is the recall for the combined AD and LBD group.

In addition to the abovementioned tests, another test named T1WML_*riu*2_ was applied to assess whether the classification performance would differ when rotational invariant LBP were calculated alone or in combination with selection of uniform LBP values only.

Total accuracy is generally higher in the T1 case (ranging from 0.97 (0.04) to 0.98 (0.04)) compared to the FLAIR case (0.80 (0.12)) but approximately similar to the two different ROIs when T1 MR images are used. Precision for class 0 is higher in the case of LBP and *C* calculated in the WML area of the T1 image (0.98 (0.06)) as compared to all of the WM area (0.96 (0.08)). Recall for class 0 is similar for both ROIs. This is also the case for precision for class 1 (0.99 (0.04)), but recall for class 1 is higher when LBP and *C* are calculated in the WML region 0.99 (0.05) as compared to the WM region (0.97 (0.06)).

When the rotational invariant calculation of LBP is combined with selection of the uniform values only, the *P*0 and *R*1 are similar to the *ri*-case. The *riu*2-case had marginally higher values for total accuracy, *P*1, and *R*0.

For comments on the T1WML_svg,*ri*_ test, see Section 6.4.

### 6.3. Two-Class Problem: AD versus LBD

Results for the two-class problem with class 1 being AD and class 2 being LBD are shown in detail in Table 4.

Classification performance was highest in the T1 case when WM was used as ROI.

### 6.4. Stavanger Data Only

In both the three-class problem and the two-class problem, NC versus AD + LBD, a fifth test was run, named T1WML_svg,*ri*_, which indicates that the T1 MR images were used for calculation of the LBP and *C*, that the WM was the ROI, and that only data from the MR scanner located at Stavanger University Hospital were used. This experiment was done to assess the robustness of the method. The rotational invariant variant of the LBP feature was used in this test. An even better performance was reached in both cases. In the three-class problem, a total accuracy of 0.91 (0.15) was achieved and all of the cases in the data set were classified correctly in the two-class problem. An implication of this is that between-centre noise falsely reduces classification accuracy and that the developed method shows even higher performance when all data come from the same scanner.

## 7. Discussion

Our results improved doing LBP texture analysis in 3DT1 image rather than the FLAIR image, indicating that there exists more textural information in the 3DT1 image compared to the FLAIR image relevant to our problem formulation. In the three-class problem as well as in the two-class problem NC versus AD + LBD, our results indicate that there exists similar amount of relevant textural information regarding dementia classification using all of WM as ROI compared to using only WML. This could be a benefit. WML segmentation is unsatisfactorily developed and very often demanding manual outlining is required as well as a FLAIR MR image, where WML is hyperintense, while WM segmentation is readily available from many well known and freely downloadable software packages needing only a 3DT1 MR image which is a common part of a clinical MR protocol. In addition, recent focus on diffusion tensor imaging (DTI) in vascular disease [58], amnestic mild cognitive impairment (aMCI) [59], and dementia [60–62] strengthens the view that age-related changes in WM play an important role in the development of dementia. DTI is, nevertheless, not sufficiently available and at the same time is costly making other approaches for WM analysis, like ours, a valuable addition.

In the two-class problem, AD versus LBD, we did not reach a comparable classification result compared to the AD + LBD versus NC case. There probably exist several explanations for that, one of the most obvious being the small sample size in the LBD class compared to the other classes. The LBD subjects are mainly classified as AD subjects indicating that the two groups experience similarities concerning our methods. Even though the two groups show different neurological etiologies, they do not differ equally regarding vascular changes. Having few subjects in the LBD group, the calculated texture features may not represent the group with proper specificity or generality. Another explanation could be related to the common basis for neurodegenerative dementias pointed out by Bartzokis in [21] or Schneider's observations about mixed brain pathologies in dementia [63].

In the three-class problem, NC versus AD versus LBD, the accuracy for the LBD class is improved showing a precision of 0.85 (0.11) and recall of 0.78 (0.20). When doing the same test on the data from Stavanger only, even better results were achieved with a precision of 0.87 (0.22) and a recall of 1.00 (0.00) for the LBD class. Vemuri et al. [18] used atrophy maps and a *k*-means clustering approach to diagnose AD with a sensitivity of 90.7% and a specificity of 84%, LBD with a sensitivity of 78.6% and specificity of 98.8%, and FTLD with a sensitivity of 84.4% and a specificity of 93.8%. A strength of their study was that they only used MR images of later histologically confirmed LBD patients. They also report sensitivity and specificity for the respective clinical diagnoses. AD with a sensitivity of 89.5% and a specificity of 82.1%, LBD with a sensitivity of 70.0% and specificity of 100.0%, and FTLD with a sensitivity of 83.0% and a specificity of 95.6%. Compared to the reported sensitivity and specificity for clinical diagnosis, our method shows substantial higher accuracy for LBD and comparable accuracy for AD. A limitation is the use of different measures of goodness to the classification results and that different data is used. In Kodama and Kawase [40], a classification accuracy of 70% for the LBD group from AD and NC is reported. Burton et al. report a sensitivity of 91% and a specificity of 94% using calculations of medial temporal lobe atrophy assessing diagnostic specificity of AD in a sample of patients with AD, LBD, and vascular cognitive impairment but do not report results for the LBD group [17]. In [19], Lebedev et al. use sparse partial least squares (SPLS) classification of cortical thickness measurements reporting a sensitivity of 94.4 and a specificity of 88.89 discerning AD from LBD.

To verify that the classification results are not driven by differences in the local variation of signal intensities (the *C* values) between centres used during collection of MR data in the study, the test T1WML_svg,*ri*_ was conducted on the Stavanger data only. The results showed an increase in classification performance, which gives us reason to believe that the results reflect real diagnostic differences.

LBP is based on local gradients and is therefore prone to noise and could be a limitation to our approach. LBP values calculated in a noisy neighbourhood would be recognised by many transitions between 0s and 1s. We performed a test, the T1WML_*riu*2_ test, where only rotational invariant and uniform LBP values, showing a maximum of two transitions between 0s and 1s, are collected. The result showed identical results as the T1WML_*ri*_ test indicating that noise does not constitute a severe problem in our method. Even though noise reduction procedures can be useful in the application of, for example, segmentation, a noise reduction approach could remove relevant textures. The contrast measure is invariant to shifts in gray-scale but not invariant to scaling. We do not use any normalization of the images prior to the feature calculation. Thus, one could argue that different patients are scaled differently making the contrast measure less trustworthy. On the other hand, if a normalization is done, for example, based on a maximum intensity value, this could indeed change the local subtle textures and affect the contrast measures, possibly in a negative way. In the present work, we have investigated the discriminating power of the features calculated without any smoothing or normalization, since the effect of such operators is not clear for this application. In future work, we want to investigate the use of different preprocessing steps, using both denoising and normalization, and compare the discrimination power of the features with and without preprocessing. The improvement in results when using data from one centre only (Stavanger) indicates lack of robustness which can be related to the facts mentioned above.

As mentioned in Section 2.2, Cronbach's alpha was calculated using total brain volume to ensure that our data material was consistent even though it was collected from different centres spanning a time scale. Texture features can be exposed to noise and a limitation to our study is the lack of using texture features for the reliability analysis.

Another limitation to our study is the lack of clinical interpretation of texture features which is difficult in our case, since brain regional information is lost in the process of feature calculation.

### 7.1. Conclusion

This study demonstrates that LBP texture features combined with the contrast measure *C* calculated from brain MR images are potent features used in a machine learning context for computer based dementia diagnosis. The results discerning AD + LBD from NC are especially promising, potentially adding value to the clinical diagnose. In the three-class problem, the classification performance exceeded the accuracy of clinical diagnosis for the LBD group, at the same time keeping the classification accuracy for the AD group comparable to the clinical diagnoses. A lower accuracy was achieved when classifying AD from LBD in the two-class problem, AD versus LBD. We considered it good news that the results using WM as ROI gave almost equally good classification performance as WML, since the WM segmentation routine is much more accessible compared to WML segmentation. The performance using 3DT1 images for texture analysis was notably better than when using FLAIR images, which is an advantage, since most common MR protocols include a 3DT1 image.

For future work we will look into texture features calculated in a 3D neighbourhood. 3D texture features have shown to be an important step towards better discrimination in machine learning systems when the images are intrinsic three-dimensional like many MR sequences are [64]. In addition, we will perform correlation analysis between texture features and cognition, since that could improve the clinical value of our work.

## Figures and Tables

**Figure 1:**
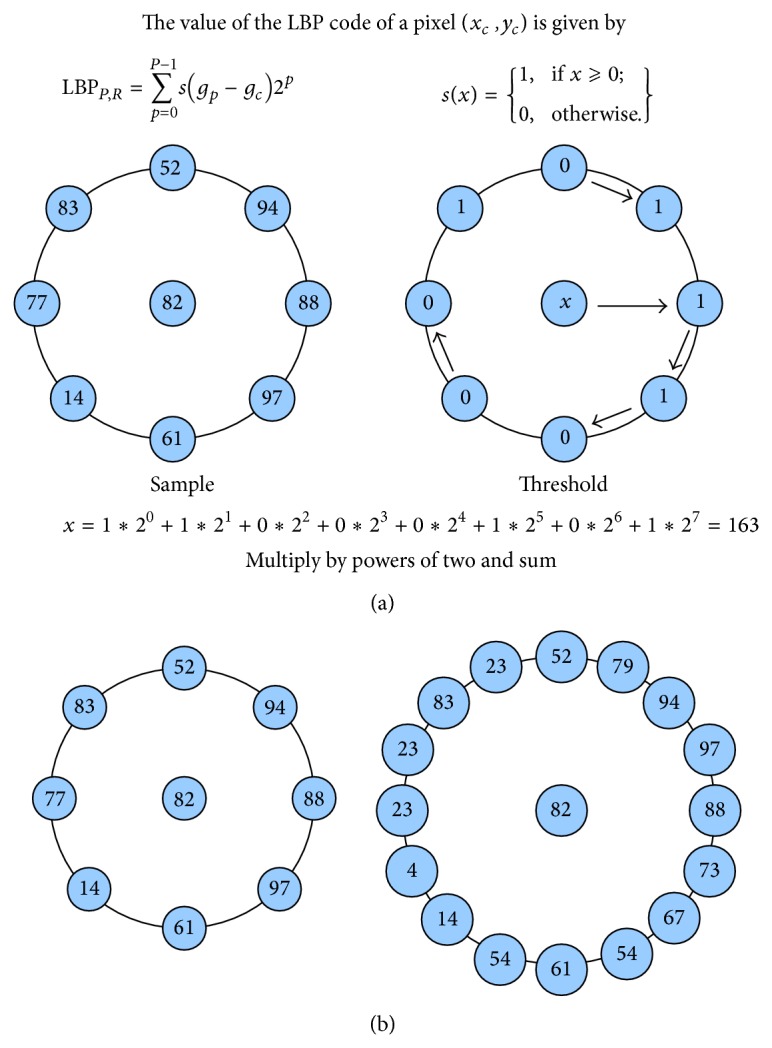
(a) Demonstrates LBP thresholding. A neighbour to a center pixel is set to one if it has equal or higher pixel value and zero if it has lower pixel value. In an anticlockwise manner, every neighbour is multiplied by powers of two and summed as demonstrated in (9). (b) demonstrates how the radius and number of samples can be varied in the choice of neighbourhood. (b) Left figure with small radius and 8 samples. Right figure with large radius and 16 samples.

**Table 1:** 

	Actual positive	Actual negative
Predicted positive	TP	FP
Predicted negative	FN	TN

**Table 2:** Results are reported as mean with standard deviation in brackets, *m*(*s*), over 10-fold cross validation, classifying NC versus AD versus LBD. *T* = total accuracy, *R* = recall, and *P* = precision. 0 for class NC, 1 for class AD, and 2 for class LBD. ROI is either WM for white matter or WML for white matter lesion area.

Test	*T*	*P*0	*P*1	*P*2
		*R*0	*R*1	*R*2
FLAIR-WML_*ri*_	0.60 (0.13)	0.71 (0.28)	0.61 (0.14)	0.33 (0.41)
		0.48 (0.25)	0.77 (0.28)	0.20 (0.35)

T1WML_*ri*_	0.82 (0.12)	0.96 (0.10)	0.80 (0.11)	0.58 (0.49)
		0.98 (0.08)	0.88 (0.18)	0.25 (0.35)

**T**1**W** **M** **L**_**ri**_^**SMOTE**^	**0.87 (0.08)**	0.97 (0.07)	0.81 (0.17)	0.85 (0.11)
		1.00 (0.00)	0.82 (0.16)	0.78 (0.20)

T1WM_*ri*_	0.82 (0.09)	0.96 (0.08)	0.81 (0.11)	0.42 (0.49)
		1.00 (0.00)	0.88 (0.16)	0.20 (0.35)

T1WM_*ri*_^SMOTE^	0.75 (0.13)	0.90 (0.12)	0.66 (0.16)	0.70 (0.21)
		1.00 (0.00)	0.72 (0.19)	0.55 (0.22)

T1WML_svg,*ri*_^SMOTE^	0.91 (0.15)	1.00 (0.00)	1.00 (0.00)	0.87 (0.22)
		1.00 (0.00)	0.77 (0.42)	1.00 (0.00)

**Table 3:** Results are reported as mean with standard deviation in brackets, *m*(*s*), over 10-fold cross validation, classifying NC versus AD + LBD. *T* = total accuracy, *R* = recall, and *P* = precision. 0 for class NC and 1 for class AD + LBD. ROI is either WM for white matter or WML for white matter lesion area.

Test	*T*	*P*0	*P*1
		*R*0	*R*1
FLAIR-WML_*ri*_	0.80 (0.12)	0.69 (0.20)	0.87 (0.11)
		0.72 (0.23)	0.84 (0.12)

**T**1**W** **M** **L**_**ri**_	**0.98 (0.04)**	0.98 (0.06)	0.99 (0.04)
		0.98 (0.08)	0.99 (0.05)

T1WM_*ri*_	0.97 (0.04)	0.96 (0.08)	0.99 (0.04)
		0.98 (0.08)	0.97 (0.06)

T1WML_*riu*2_	0.98 (0.04)	0.96 (0.08)	1.00 (0.00)
		1.00 (0.00)	0.97 (0.06)

T1WML_svg,*ri*_	1.00 (0.00)	1.00 (0.00)	1.00 (0.00)
		1.00 (0.00)	1.00 (0.00)

**Table 4:** Results are reported as mean with standard deviation in brackets, *m*(*s*), over 10-fold cross validation, classifying AD versus LBD. *T* = total accuracy, *R* = recall, and *P* = precision. 1 for class AD and 2 for class LBD. ROI is either WM for white matter or WML for white matter lesion area.

Test	*T*	*P*1	*P*2
		*R*1	*R*2
FLAIR-WML_*ri*_	0.73 (0.15)	0.78 (0.11)	0.20 (0.45)
		0.91 (0.12)	0.10 (0.32)

T1WML_*ri*_	0.66 (0.17)	0.74 (0.10)	0.00 (0.00)
		0.84 (0.18)	0.00 (0.00)

T1WML_*ri*_^SMOTE^	0.73 (0.16)	0.72 (0.18)	0.76 (0.17)
		0.75 (0.20)	0.71 (0.19)

T1WM_*ri*_	0.74 (0.16)	0.80 (0.09)	0.45 (0.51)
		0.75 (0.20)	0.71 (0.19)

T1WM_*ri*_^SMOTE^	0.68 (0.14)	0.67 (0.14)	0.75 (0.21)
		0.69 (0.29)	0.68 (0.14)
